# Patterns and barriers of mental health service utilization among medical students: a cross-sectional study

**DOI:** 10.1186/s43045-022-00267-0

**Published:** 2022-12-15

**Authors:** Doaa Abdel-Hady, Mohamed Baklola, Mohamed Terra, Abdel-Hady El-Gilany

**Affiliations:** 1 Public Health and Community Medicine Department, Faculty of Medicine, Mansoura University, 60El-Gomhoria Street, Mansoura, 35516 Egypt; 2 Faculty of Medicine, Mansoura University, 60El-Gomhoria Street, Mansoura, 35516 Egypt

**Keywords:** Mental health, Medical students, Patient acceptance of health care, Mental health services, Health behavior

## Abstract

**Background:**

The prevalence of mental health problems among medical students has been steadily rising. It is greater than the prevalence of mental health problems among other students, negatively impacting students, and their future careers. The study aims to estimate the prevalence of the self-reported need for mental health care, the pattern of utilization of mental health services, and the different barriers that hinder medical students from seeking professional help.

**Results:**

This study was conducted among medical students at Mansoura University, using a structured self-reported online questionnaire to collect the need for mental health services, sociodemographic details, the pattern of utilization of mental health services, and the barriers using a Likert scale of 30 items named Barriers to Access to Care Evaluation Version 3. According to this study, 77.77% felt the need for mental health care. The independent predictors for feeling the need for mental health care were female sex and urban residence with an adjusted odds ratio of 2.7 and 1.9, respectively. Regarding mental healthcare needs, most of the barriers were instrumental and attitudinal related. Lack of information about how to access services and solve the problem by themselves was the most common barriers followed by time and financial affords.

**Conclusions:**

It appears that Mansoura medical students are at higher risk of feeling the need for mental care. Considerable barriers to help-seeking remain prevalent, including both logistical (e.g., time) and informational (e.g., lack of knowledge about the available services).

## Background

There is cumulative evidence of the increasing prevalence of mental illness among different age groups. However, a substantial proportion of people with mental illness do not receive care [1]. In Africa, only 14 out of every 100,000 people with mental health problems receive treatment [2]. This is a clear representation of the unfavorable attitude toward mental health care. Mental disorders are common in medical students and represent a major challenge in the medical training of future physicians. Several studies showed that medical students experience high levels of depression, stress, and burnout compared to students pursuing other professional courses and the general population [3, 4]. Some studies have suggested that mental health issues among medical students might adversely influence their academic performance, competency, and professionalism, contribute to academic dishonesty, and play a role in alcohol and substance abuse [5].

A previous study in Egypt found that approximately more than half of students had one or more mental disorders. The prevalence of high Positive Symptom Distress Index (PSDI), depression, and somatization was 30.1%, 25.2%, and 21.7%, respectively [6]. Medical education is demanding, and the intense environment creates excessive pressure on medical students’ psychological well-being which can have unfavorable effects personally or professionally [7]. The reported stressors for medical students appear to be associated with the medical curriculum [8].

Despite this high prevalence of mental illness among medical students, only a small percentage of medical students seek professional help or guidance for mental health problems [9]. This may reflect a lack of awareness of mental health conditions or the available treatments. Furthermore, 51% of medical students sought informal help, and only 18% sought professional help. However, it is known that seeking professional help is the first step to achieving a better life, but it is not accessible due to various barriers [10]. So, it is necessary to study and understand the various barriers that hinder students from seeking help.

There is a lack of an inclusive overview to assess the needs and barriers to mental health care among medical undergraduates in Egypt to the best of the authors’ knowledge. Consequently, the objectives of the study are to describe the prevalence of the self-reported need to seek mental health services, the pattern of utilization of mental health services, and the different barriers that hinder medical students from seeking professional help.

## Methods

This is a cross-sectional descriptive study with an analytic component, carried out in the Faculty of Medicine, Mansoura University, Egypt, during the academic year 2020–2021. This study targeted undergraduate medical students who were willing to participate voluntarily in the study. The primary outcome of interest is the prevalence of medical students who felt a need for mental health care and based on the reported rate of 44.6% from a previous study [10], considering an alpha error of 5%, study power of 80%, 5% precision, using MDCalc 15.8, and the sample size is 359 students. Five-hundred and fifty-eight students successfully submitted a full response.

The questionnaire included four sections: sociodemographic, a single question to collect the self-reported feeling about the need to seek mental health care, a single question to describe the pattern of utilization of mental health services, and a structured self-reported online English scale that was used to define the barriers hindering the students from seeking mental health services. The barriers to seeking professional care were assessed using a Likert scale of 30 items named Barriers to Access to Care Evaluation (BACE) Version 3 which demonstrates acceptable levels of reliability and validity [11].

Six items were removed from the scale as instructed by the (BACE) scale as they were not applicable to student-level participants as they were asking about employment and children-related barriers, which is not suitable for our study setting. BACE consists of three subscales: stigma, attitudinal, and instrumental-related barriers. Each response was ranked from 0 to 3 as follows: 0 — not at all, 1 — quite a little, 2 — quite a lot, and 3 — a lot. In the analysis part, we considered 0 — not at all as not being a barrier, 1 — quite a little as a minor barrier, 2 — quite a lot, and 3 — a lot as a major barrier.

The questionnaire was available on Google Forms from September 1, 2020, until the sample was fulfilled. We posted it through the official groups of all the academic years on the Telegram application in order to reach all the students with no obligation to fill out the questionnaire. They were also allowed to respond in their own time and anonymously.

### Statistical analysis

Data were analyzed using the Statistical Package for Social Science Program (SPSS 25 for Windows). Quantitative data were summarized as a mean ± standard deviation and median for nonparametric data. For the qualitative data, we used numbers and percentages and analyzed them using the Chi-square test and crude odds ratios (COR), and their 95% confidence interval was calculated. Adjusted logistic regression analysis was done using the forward Wald method to detect independent predictors of the need for mental health care. Mann-Whitney was used to compare variation in the total scale and its subscales with different parameter studies. We used Pearson’s correlation coefficient to examine the correlation, as well. *P*-value ≤ 0.05 was considered statistically significant.

## Results

The questionnaire was answered by 558 medical students with females making up the majority (59.5%). More than two-thirds (77.8%) of the participants had a feeling of the need to seek mental health care. In terms of residence, more than half (54.3%) were from rural areas. Moreover, the medical students from preclinical years were making up the vast majority 79.4% (Table 1). The independent predictors for feeling the need for mental health care were female sex and urban residence with adjusted odds ratio of 2.7 and 1.9, respectively. There was no significant difference between sex, academic stage, and residence regarding seeking professional help among those who felt the need for seeking mental healthcare services.

**Table 1:** Independent predictors for feeling the need to seek mental healthcare and actual seeking of professional care

	Total	*N* (%)	*P*-value	OR (95% *CI*)	Adjusted OR (95% *CI*)
**Feeling the need to mental health care**					
Sex					
Male	226	154 (72.5%)	<0.001	1 (*r*)	1 (*r*)
Female	332	280 (84.3%)		2.5 (1.7–3.7)	2.7 (1.8–4.1)
Academic stage					
Pre-clinical	393	297 (75.57%)	0.58	1 (*r*)	
Clinical	165	137 (83.3%)		1.6 (0.99–2.5)	
Residence					
Urban	255	209 (82%)	0.029	1.6 (1–2.4)	1.9 (1.2–2.9)
Rural	303	225 (74.3%)		1 (*r*)	1 (*r*)
Overall	**558**	**434 (77.8%)**			
**Seeking professional help among who felt the need for mental health care**					
Sex					
Male	154	32 (20.8%)	0.205	1 (*r*)	
Female	280	68 (24.3%)		1.22 (0.76, 1.96)	
Academic stage					
Pre-clinical	297	63 (21.2%)	0.093	1 (*r*)	
Clinical	137	37 (27.0%)		1.374 (0.86, 2.19)	
Residence					
Urban	209	52 (23.1%)	0.191	1.22 (0.78, 1.91)	
Rural	225	48 (23.0%)		1 (*r*)	
Overall	**434**	**100 (23%)**			

Despite their need for mental health care, 64.3% of the students did not seek it. Furthermore, 12.7% sought informal help, while just 23% sought professional help (data not shown in tables).

Table 2 shows that the median for the total stigma score was significantly higher in the preclinical stage than in the clinical stage, for the total instrumental score, and the median was significantly higher in female sex than male, rural than urban, as well. There were no significant differences between other factors.

**Table 2:** Variation of total scale and different subscales of (BACE) score according to sex, academic stage, and residence

	Sex		Academic stage		Residence	
	Male	Female	Preclinical	Clinical	Urban	Rural
Total stigma score Median (Q1-Q3)	6 (2.75–10)	6 (2–10)	6 (3–11)	5 (2–9)	6 (3–10)	6 (2–10.5)
***p***	**0.8**		**0.004**		**0.7**	
Total attitudinal score Median (Q1-Q3)	8 (5–10.25)	8 (6–11)	8 (6–11)	8 (6–10)	8 (6–11)	8 (6–11)
***p***	**0.3**		**0.06**		**0.9**	
Total instrumental score Median (Q1-Q3)	8 (5–11)	9 (6–12)	9 (6–11)	8 (5–11)	8 (5–10)	10 (6–12)
***p***	**0.001**		**0.09**		**0.001**	
Total score Median (Q1-Q3)	21 (16–28.2)	24 (18–30)	24 (18–30)	21 (16–27)	23 (17–28)	24 (17.5–30)
***p***	**0.05**		**0.003**		**0.2**	

Table 3 shows the barriers that hinder medical students from seeking professional health care. The top two barriers were “I am not sure where to go to get professional care” and “I want to solve the problem on my own” by 61.29% and 55.99%, respectively.

**Table 3:** Barriers to Access to Care Evaluation (BACE) as perceived by medical students

	Not a barrier (not at all stopped me)	Minor barrier (stopped me a little)	Major barrier (stopped me quite a lot & a lot)	Mean (SD)
I am concerned that I might be seen as weak for having a mental health problem?	177	156	101	0.82 (0.782)
I am concerned about what my family might think?	134	113	187	1.12 (0.852)
I am concerned that I might be seen as “crazy”?	276	78	80	0.55 (0.786)
I don’t want a mental health problem to be on my medical records?	207	99	128	0.82 (0.861)
I am concerned that people I know might find out?	205	108	121	0.81 (0.846)
I am concerned that people might not take me seriously if they found out I was having professional care?	202	94	138	0.85 (0.874)
I am concerned about what my friends might think, say, or do?	241	99	94	0.66 (0.812)
I feel embarrassed or ashamed?	181	127	126	0.87 (0.832)
**Total stigma score**				**6.51 (4.688)**
**Total stigma score (median, Q1-Q3)**				**6 (2–10)**
I dislike talking about my feelings, emotions, or thoughts?	118	92	224	1.24 (0.854)
I want to solve the problem on my own?	93	98	243	1.35 (0.810)
I am concerned about the treatments available (e.g., medication side effects)?	117	95	222	1.24 (0.851)
I am afraid of being put in hospital against my will?	244	66	124	0.72 (0.879)
I think that professional care probably would not help?	167	122	145	0.95 (0.847)
I think I did not have a problem?	186	126	122	0.85 (0.830)
I prefer to get help from family or friends?	235	76	123	0.74 (0.872)
I prefer to get alternative forms of care (e.g., traditional/religious healing or alternative/complementary therapies)?	190	88	156	0.92 (0.890)
I have had previous bad experiences with professional care for mental health?	350	39	45	0.30 (0.646)
**Total attitudinal score**				**8.32 (3.639)**
**Total attitudinal score (median, Q1-Q3)**				**8 (6–11)**
I feel too unwell to ask for help?	138	119	177	1.09 (0.848)
I cannot afford the financial costs?	105	89	240	1.31 (0.837)
It is difficult to take time off work or study?	93	107	234	1.32 (0.806)
I have problems with transport or travelling to appointments?	166	107	161	0.99 (0.869)
I am not sure where to go to get professional care?	80	88	266	1.43 (0.784)
I don’t have anyone who could help me get professional care?	105	99	230	1.29 (0.831)
Professionals from my own ethnic or cultural group not being available?	195	92	147	0.89 (0.882)
**Total instrumental score**				**8.32 (3.628)**
**Total instrumental score (median, Q1-Q3)**				**8 (6–11)**
**Total score**				**23.15 (8.792)**
**Total score (median, Q1-Q3)**				**23 (17–29)**

Regarding stigma-related barriers, the most reported barrier was “I am concerned about what my family might think,” rated 43%, followed by “I felt embarrassed or ashamed,” rated 29%. For attitudinal-related barriers, the two were “I want to solve the problem,” rated 55.99%, and “I dislike talking about my feelings, emotions or thoughts,” rated 51.62%. For instrumental-related barriers, “I am not sure where to go to get professional care” rated 61.29%, “I cannot afford the financial costs” rated 54.54%, and “It is difficult to take time off work or study” rated 53.9% were the most three barriers.

The correlation matrix in Table 4 demonstrates that the entire subscales had a highly significant correlation with one another. Total attentional score with total instrumental score showed the highest correlation between all the subscales with (*r* = 0.32). Regarding their correlation to the total score, stigma was the highest (*r* = 0.78).

**Table 4:** Correlation between total score and each subscale score

	Total stigma r (*P*)	Total attitudinal r (*P*)	Total instrumental r (*P*)
Total attitudinal score	0.303 (≤ 0.001)		
Total instrumental score	0.297 (≤ 0.001)	0.321 (≤ 0.001)	
Total score	0.781 (≤ 0.001)	0.708 (≤ 0.001)	0.704 (≤ 0.001)

## Discussion

According to this study, 77.77% of medical students have reported feeling the need for mental health care. This is much higher than the reported prevalence of 44.6% among medical students in India [10]. However, this high prevalence can be referred to as the drastic effect of the COVID-19 pandemic on all sectors of the community [12], especially medical students, who have greater worry about their future and the perceived pressure from their social circle as a source of information and care. More importantly, they fear transmitting the infection to their family that they may have acquired during their medical training and exams.

In addition to this, the loss of loved ones and the transition to online education may both contribute to an increase in levels of stress and anxiety. According to the findings of a longitudinal study that was carried out in China, acute stress, anxiety, and depressed symptoms were rather common among college students during the COVID-19 pandemic. Furthermore, these symptoms exhibited a considerable rise beyond the first stage of the outbreak [13].

Furthermore, females are also 2.7 times more likely than males to feel the need for mental health care. In contrast to a meta-analysis study that includes cross-sectional studies that investigated the distribution of any mental health disorder among Chinese medical students [14], there was no significant difference in gender regarding mental health needs. This significant difference could be due to increased social-cultural pressure on females during their academic years, which puts a tremendous burden on them.

Based on student residence, there is a significant difference between urban and rural students regarding feeling the need to seek mental health care with a probability of 1.9 times more likely in urban students than rural ones. This could be due to loneliness, violence, high crime rates, homelessness, noise and other pollution, traffic accidents, and drug misuse which prevail more in urban regions [15]. On the other hand, in rural areas, the traditional joint family and warm neighborhood may be considered a source of high social support, and it may buffer perceived stress through a support network promoting mental health [16]. However, urban students have higher access to mental health services as the median instrumental total score is significantly lower than rural, in accordance with a previous study [17].

Despite their need for mental health care, only 23% sought professional help. Although this is a lower rate, it is consistent with some studies that indicated varied rates of professional help utilization of 18% and 22.7% in India and the USA, respectively [10, 17]. Some medical schools in the USA, on the other hand, had a higher rate of 42.1% and 33% [18, 19].

Depending on the study results, most of the barriers were instrumental and attitudinal related. Other studies depict that most reported barriers were related to attitude and stigma [10, 20]. While stigma was the highest barrier in some studies [10, 18], our findings identified stigma as the lowest barrier, thus indicating that students are aware of mental health illnesses and no longer ashamed of having such conditions.

Also, all the subscales in this study had a statistically significant correlation with one another (*P* < 0.001), and this is in accordance with a study that investigated these correlations in India [10]. This is since stigma may have a significant impact on the public’s attitudes toward mental health treatment. For example, the urge to resolve the problem independently, even though it had a high score, could be related primarily to stigma. Likewise, the lack of resources may contribute to opposition to seeking mental health care or an unfavorable outlook toward expert help.

Reported barriers to professional help were different in numerous studies, although the absence of knowledge about accessibility to mental health services and solving the problem by themselves was the most common barriers to care reported by our students, followed by time and financial affords. In Kasam [10], the most prominent one was lack of time, followed by uncertainty about where to find professional help. As reported by Rodriguez [19], convenience, stigma, and concerns about confidentiality were the most common barriers. In Ebert [21], the preference to handle the problem alone, followed by wanting to talk with friends or relatives instead, was rated the most important. And according to Menon [22], lack of time and being unaware as to where to seek formal help were the most common barriers.

So, a continuous effort to improve the accessibility and acceptance of mental health services early in their careers is crucial. Fortunately, the most commonly reported barrier in this study is the lack of information which is the easiest to overcome, and this emphasizes the need of educating students about the available mental health care and where to access it. This can be achieved simply by organizing a committee which concerns about how to access care and raising awareness about mental health problems. Moreover, this educational approach will cover more barriers such as financial costs and the advantages of the services available to the students. Also, the significant positive correlation between all subscales implies that the decrease of instrumental barriers mentioned above would also contribute to the decline of other barriers and vice versa.

## Conclusions

Our study is considered the first of its kind in Egypt to describe the barriers to mental health care for undergraduate medical students. It provides another step in assessing medical students’ utilization of mental health services and identifying barriers to care, including instrumental related to mental health services.

While it appears that Mansoura medical students are more likely than their peers to need mental care, considerable barriers to help-seeking remain prevalent, including both logistical (e.g., time) and informational (e.g., lack of knowledge about the available services).

### Limitations of the study

This study has some limitations: first, few socioeconomic and demographic data were collected to ensure students felt confident and their responses would remain confidential. Second, the results are potentially susceptible to self-selection bias and can also be affected by the COVID-19 impact on our participant’s mental health, so we suggest that further studies should be conducted to assess the prevalence of the need and barriers post COVID-19. Finally, the findings are based on data collected from a single medical school, and the generalizability of these results to other medical schools is unclear.

## Data Availability

The datasets used during the current study are available from the corresponding author upon request.

## Abbreviation

BACE: Barriers to Access to Care Evaluation
